# AI Techniques for COVID-19

**DOI:** 10.1109/ACCESS.2020.3007939

**Published:** 2020-07-08

**Authors:** Adedoyin Ahmed Hussain, Ouns Bouachir, Fadi Al-Turjman, Moayad Aloqaily

**Affiliations:** 1 Department of Computer EngineeringNear East University 52988 99138 Nicosia Mersin 10 Turkey; 2 Department of Computer EngineeringZayed University 54483 Dubai United Arab Emirates; 3 Research Centre for AI and IoTDepartment of Artificial Intelligence EngineeringNear East University 52988 99138 Nicosia Mersin 10 Turkey; 4 College of EngineeringAl Ain University Al Ain United Arab Emirates; 5 College of Technological InnovationZayed University 54483 Dubai United Arab Emirates

**Keywords:** Big data, the IoT, artificial intelligence, cloud computing, deep learning

## Abstract

Artificial Intelligence (AI) intent is to facilitate human limits. It is getting a standpoint on human administrations, filled by the growing availability of restorative clinical data and quick progression of insightful strategies. Motivated by the need to highlight the need for employing AI in battling the COVID-19 Crisis, this survey summarizes the current state of AI applications in clinical administrations while battling COVID-19. Furthermore, we highlight the application of Big Data while understanding this virus. We also overview various intelligence techniques and methods that can be applied to various types of medical information-based pandemic. We classify the existing AI techniques in clinical data analysis, including neural systems, classical SVM, and edge significant learning. Also, an emphasis has been made on regions that utilize AI-oriented cloud computing in combating various similar viruses to COVID-19. This survey study is an attempt to benefit medical practitioners and medical researchers in overpowering their faced difficulties while handling COVID-19 big data. The investigated techniques put forth advances in medical data analysis with an exactness of up to 90%. We further end up with a detailed discussion about how AI implementation can be a huge advantage in combating various similar viruses.

## I. Introduction

Enthusiasm for artificial intelligence (AI) has been high for as far back as hardly any years, arriving at the top in 2018. This promotion is because of the critical advances in the field of AI and the broad applications in reality [1]. AI procedures have sent enormous waves across restorative administrations, on any occasion, fuelling a working conversation of whether AI specialists will as time goes on supersede human experts later on. At any rate, AI helps authorities by choosing good medical choices or at most supersede judgments by humans in a particular field of a general advantage like radiology. However, with the developing accessibility of general prosperity of information and the energetic improvement in the monstrous information, expressive methods have made the advancing profitable uses of this AI technology conceivable. Guided by critical clinical solicitations, essential AI strategies can open medically material data concealed in the massive extent of information, which along these lines drives effectively [2] [3]. Affirmed instances of the coronavirus infection (COVID-19) surpass those with extreme intense respiratory disorder (e.g., SARS). By correlation, SARS confirmed death case is 774 individuals in 2003. Both COVID-19 and SARS spread across landmasses, taint creatures, and people and utilize comparative mechanics to enter and contaminate the cell [4]. On the cutting edge, a strategic reaction to COVID-19 is like that of SARS yet one significant contrast exists. In a long time since SARS, an amazing new device has been developed that might be instrumental in keeping this infection inside sensible cut-off points in particular. It is the man-made brainpower (AI). Few would contend that AI is causing a change in general health and there may be an incentive in the use of AI to the current COVID-19 episode. For instance, in anticipating the area of the following flare-up [5]. This application is successfully what the Canadian organization, Blue Dot, has endeavored to do and in that capacity was generally announced as the main association to uncover updates on the episode in late December. Different utilizations of AI that have risen in light of the most recent pandemic incorporate Benevolent AI and Imperial College London, which report that a medication affirmed for rheumatoid joint inflammation, baricitinib, may be viable against the infection. Meanwhile, Insilico Medicine situated in Hong Kong as of late declared that it’s AI calculations had planned six new atoms that could end viral replication [6].

Because medical information continues to improve rapidly, medical data has grown quickly and huge numbers of medical devices have created an incredible risk on current hospital information systems [7]. The field of healthcare generates a wide range of data on medical diagnosis, patient records, treatment, medication, diagnosis, etc. The real concern is that because of the limited data processing, the accuracy of certain reports has an impact on the organization of healthcare [8]. From that issue, the data is useless without an accurate result which has been produced from the processing of the huge data. Using the artificial intelligence can improve the result by employing systems that collect hundreds of actual patient information and examine it by experts and artificial intelligence tools [9]. Big data in healthcare sectors are already applied in some hospitals, especially in the field of radiology by using advanced algorithms such as Deep Learning [10]. Big data visualization plays an important role also not only in the management of the hospital but also in reducing the medical waste [11]. Simultaneously, various organizations in France have indicated their enthusiasm for the utilization of AI in medicinal services. Therefore, as indicated by key supposition pioneers, a few claims to fame could be completely replaced by AI gadgets, prompting an expert change for the influenced doctors, with regards to the adamant strengths which is pathology, and also radiology [12]. Hence, some countries like France have a portion of these pros that are good and that they adapt to numerous fast changes like this, as in numerous different nations [13], [14]. Although the USA is topping the front line, France, presently attempts to meet up with speed, going to the massive measure of wellbeing information assembled by the organization and open administrations at the National System of Health Data situated in France [15]. However, measures of information inside the framework continue expanding also it as of now incorporates the information from repaid social insurance counting medical coverage information, the clinical reason for death (CépiDC), handicap information gotten at Independent-living support fund, with an example of information by strengthening medical coverage associations. Moreover, after reports [16], the president of France, declared the formation of a “Wellbeing Data Hub” with a solid purpose of France worldwide AI system. It is confided in outsiders between wellbeing information makers, entertainers wishing to utilize such information, and residents or common help agents. The main strategic the center point will be to advance the social event of clinical information, which is the information gathered throughout care, in a mysterious way [17] [18]. Simultaneously, continuous problems come out of utilizing AI in human services, one of the principal one is the troubles with the utilization of wellbeing information, for example, information obligation, protection concerns [19], cybersecurity worries, is the subject of duty, also the coordination of AI instruments into modern enforcement and morals contemplations [20] [21]. For instance, Google as of late distributed a rundown of moral standards identified with the advancement of AI in June 2018 [22]. As of now, AI apparatuses and it is associated previously cited problems are still inside the domain of researches and portrayal, however, ordinarily, it acknowledged that the mentioned devices will upset clinical methods and clinical network is starting to pay attention to this situation [23]. In any case, medication isn’t incorporating these instruments as fast as the innovation is progressing. Also, lacking inclusion and also the participation of wellbeing experts, AI can’t be coordinated into present practice. While a specific degree of overstatement appears to have assumed control over the conversation of AI in the clinical field, we additionally found that different contemplations and information have developed among every class of partner. From one point of view specialists and scientists feature the requirement for excellent clinical information; then again, doctors are as yet sitting tight for proof of the convenience of these instruments, and miracle if they will be considered capable if there should arise an occurrence of a physical issue because of an AI apparatus that they don’t completely comprehend. In this way, in the coming years, society should be careful in regard to the spot given to huge information and AI. Moreover, since AI keeps on pushing ahead, it is up to all on-screen characters required to characterize the basic focuses for a reasonable type of clinical predictable with their qualities as we recognized right now.

### A. Comparison to Other Studies

As indicated by the meaning given by Marvin Minsky, which is known as the father of AI, it essentially implies that any machine can carry out a responsibility which is viewed as a keen one by individuals. To be sure, request for AI is the one in which the utilization falls in two classes: a) undertaking to replicate the capacities of a human cerebrum and b) arrangement of devices to do assignments that currently recommend human actions. Reproduced insight is detached into various sub-disciplines, concentrating on unmistakable issues, for example, vision, critical thinking, language appreciation, learning, and so forth. Authors in [24] said a worldview of research and a few parts of AI have become spots of multidisciplinary trade where rationalists, therapists, PC researchers, and other people who are keen on the different issues of AI can meet. Computer-based intelligence can likewise be comprehended as an idea, for example, the common and also the hypothetical view accepted the human cerebrum, and gives of a solid or extraordinary view of imagination that connects with it to relate the different discernments that it has of that view. During the Dartmouth Conference, the proposition of a plan to design AI was put forth, as an order as well as an idea as stated by authors in [25]. Authors in [26] proposed a calculation called the Normalized Maximum Likelihood (NML) technique, which consolidates substitution and measurable styles. In [27] authors proposed an improved AI model of NML having capacities of parting the arrangement into fixed-size squares and encoding them utilizing the historical backdrop of a subsequence and controlling the equivalent with Hamming separation called GeNML. The author in [28] delivered another arrangement pressure calculation called Expert Model (XM) utilizing measurable strategies. In [29] the author created GenBit Compress, a calculation that packs tedious and non-dreary successions utilizing the thoughts of broadened twofold tree. LCA, a Lossless Compression Algorithm was given in [30] for dealing with a few surmised rehashes and complimentary palindromes. A calculation, DNASC, that joins factual and substitution strategies, was proposed in [31]. The authors in [32] put forth a technique using AI tools, were affected patients with COVID-19 can be managed remotely based on self-isolation and symptomatic management. With the fast improvement of PC innovations, computerized picture handling tech have been generally implemented in the clinical field, including organ division and picture upgrade and fix, offering help for ensuing clinical findings. In [33] [34], authors proposed deep learning innovations, for example, convolutional neural system (CNN) with the solid capacity of nonlinear displaying, having broad applications in clinical picture preparing as well. In [35] [36], relevant examinations were led on the conclusion of pneumonic modules, while in [37], [38] grouping of benign and threatening tumors, [39] and aspiratory tuberculosis investigation and sickness expectation [40] around the world. The authors in [41] have emphasized the importance of the COVID-19 computed tomography (CT) qualities which can be accessible on several stages. Meanwhile, the authors in [42] have looked into six distributed examinations perceiving the clinical qualities of the COVID-19. In their work, authors have summed up these examinations and gave a concise review of clinical highlights and potential medicines for the COVID-19. Authors in [43] and [44] give a short outline of the COVID-19 episode in addition to its clinical highlights, avoidance, finding, and treatment. The essential issue with both of these works is that they audit a subset of a lot more extensive subject. Despite the fact that these studies shed some light on the current situation of the COVID-19 flare-up, they give an extremely short and constrained thoughts regarding its specific circumstances from the technical perspectives. Authors in [45] considered ninety-nine cases of the COVID-19, forty-nine of whom had an immediate connection to the Wuhan seafood market, known to be the COVID-19 focal point. Their discoveries of the epidemiological, clinical, and radiological qualities of the illness have been distributed. To decide the clinical qualities of the COVID-19, authors in [46] have contemplated on about one hundred and thirty-eight cases located in Wuhan. The authors have considered points of interest, in socioeconomics, signs and indications, and clinical history of the considerable number of patients to survey their cases cautiously. The creators have likewise introduced the research facility and discoveries of these patients to show the impacts of the SARS-CoV-2 infection on various fundamental organs of the body. In their discoveries, they report that among all the patients that were considered, 17% and 11% of cases showed intense respiratory trouble conditions and kicked the bucket of various organ brokenness disorders respectively. In spite of the robustness of exploration in the area of COVID-19 advancement, apparently, at the hour of this composition, there is no study that gives a thorough survey of the COVID-19 flare-up and its expected ramifications. Besides, no work in the current state of the art endeavors to overview advances, specifically in AI, datasets, deep learning, and cloud while dealing with the pandemic. In Table 1, we highlight these main differences and limitations in comparison to this survey.

**TABLE 1:** Comparison to Other Studies

Reference	ML	Deep learning	Algorithms	QoS	Robustness
[24]	✓		✓	✓	
[25]	✓		✓		✓
[26]		✓		✓	
[27]	✓		✓	✓	
[28]	✓		✓		
[29]	✓		✓		
[30]	✓		✓		✓
[31]			✓		✓
[32]	✓			✓	✓
[33]		✓	✓	✓	
[34]		✓	✓	✓	
[35]		✓	✓	✓	✓
[36]		✓	✓	✓	✓
[37]		✓	✓		✓
[38]		✓	✓		✓
[39]		✓	✓	✓	
[40]		✓	✓		✓
[41]	✓		✓		✓
[42]	✓	✓		✓	
[43]	✓	✓			✓
[44]	✓	✓			✓
[45]	✓	✓		✓	✓
[46]		✓	✓		✓
Our survey	✓	✓	✓	✓	✓

### B. Scope of the Survey and Contributions

The models referenced above are regions where AI is applied in checking infections like the COVID-19 which have made extraordinary accomplishments. Corresponding to the studies referenced, for the effective utilization of new AI methods, it is imperative to investigate and appreciate the troubles related to the possibility of AI and infections. At present, we will show an overview of AI as a rule of general health and put an accentuation on specific methodology and systems to be kept into thought while overseeing difficulties. One of the major upsides about AI has been comprehensively examined above in clinical organizations. Man-caused thinking can utilize refined figuring to acquire abstracts from a tremendous amount of human organization information, and a brief timeframe later utilizes the comprehension of data to help clinical implementation. Whereas, in like way be furnished in ideas and self-re-examining capabilities to advance its exactness which are dependent on investigation. The AI application can assist pros by providing bleeding-edge clinical info from papers, course books, and clinical works to advance fitting patient ideas. Also, the framework can help with diminishing definitive and remedial errors which are sure in the practitioner clinical practices. Also, an AI structure ousts critical info gotten from a colossal patient masses, to assist make advancing inducing for prosperity hazards and prosperity result prediction. In like way, our guideline contribution right now can be sketched out as follows:

• This survey gives an outline of the AI strategies joined in controlling infections.
• The techniques which empower AI frameworks to create clinically significant output have been overviewed.
• The Motivations of utilizing AI in COVID-19.
• The incorporation of cloud and edge computing against COVID-19 has been outlined.
• Diverse ML techniques against COVID-19 have been sorted.
• Moreover, we examine open research difficulties and issues.

We summarize the rest of the paper as follows. In section 2 we made an overview of the fundamentals between Artificial intelligence and COVID-19 with various learning techniques. Section 3 emphasizes on deep learning in COVID-19. In section 4 we discuss on commuting AI techniques in combating COVID-19. Also, with various AI methods including deep learning. Section 5 discusses related datasets. In section 6 we discuss performance evaluation. While in section 7, we emphasis on utilizing cloud computing servicing in combating COVID-19. Lastly, section 8 brings the end to our survey with the conclusion. Table 2 above gives a synopsis of the utilized abbreviations and their definitions.

**TABLE 2:** Used Abbreviations

Terms	Meaning
ICT	Information and Communication Technologies
XM	Expert Model
NN	Neural Networks
NML	Normalized Maximum Likelihood
LR	Linear Regression
LCA	Lossless Compression Algorithm
ML	Machine Learning
EMR	Electronic Medical Record
SVM	Support Vector Machine
CNN	Convolutional Neural System
FM	Factorization Machine
MF	Matrix Factorization
AI	Artificial Intelligence
PLMR	Personalized Multi-Linear Regression
EP	Electrophysiological
SL	Supervised Learning

## II. Artificial Intelligence and COVID-19

**FIGURE 1.**
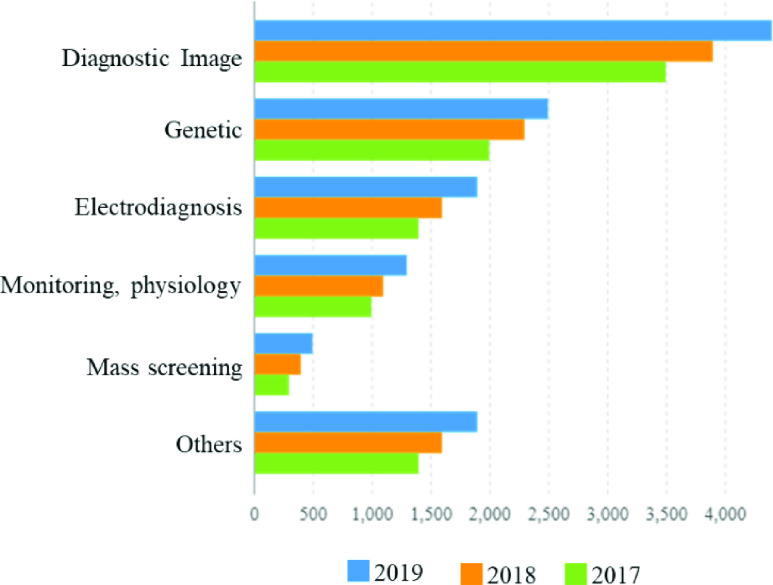
Data types considered in AI concerning COVID-19.

Before frameworks related to AI can be conveyed in COVID-19 application, they should undergo a training process through information which is produced from medical experiments, just like, screening diagnosis, virus analysis, treatment works, etc. In addition to the goal with the ability to learn comparable gatherings of objects, the relationship between objects highlights and output the premium. This clinical information regularly exists, yet never constrained to types of demographic, clinical notes, and electronic accounts from clinical gadgets, physical assessments, and clinical research facility, and images [47]. Particularly, in the finding stage, one of the significant extents of the AI data writing from determination images, also hereditary test and electrodiagnosis is illustrated in figure 1 below. Moreover, authors in [48] requested radiologists to take heed of AI advances when investigating demonstrative images which contain immense data information. In [49] they contemplated the use of unusual articulation of hereditary in distance non-coding RNAs to analyze malignant gastric growth. However, authors in [50] developed an electrodiagnosis for an emotionally supportive network in restricting brain injury.

Likewise, physical assessment jots and medical experiment center outcomes are mentioned as part of other significant information areas as shown in figure 1. Also, it depicts areas or fields where the application of AI has been of great interest in the past years. They are being recognized with a picture, also hereditary and finally electrophysiological (EP) information, just because they consist of huge segments of unstructured paper writings, for example, medical jots that are not good for analyses. As an outcome, the comparing AI applications center for first changing over the disorganized content to machine-justifiable electronic medical record (EMR). However, for instance, authors in [51] utilized AI advances to remove phenotypic highlights from case reports to improve the finding exactness of the inborn irregularities. In [52] features that Infervision’s of the application of AI limits the weight of the procedures by facilitating the judgments and observing of COVID-19. As an ever-increasing number of sweeps is done then the calculation learns and improves precision together with the infection. The estimation of AI becomes an integral factor by decreasing the weight of clinicians in a situation, for example, the present COVID-19 episode. Thinking about a quick development in the present flare-up concerning the contamination of medicinal services experts, the author in [52] calls attention to were the Infervision AI implementation can help ensure practitioners. The passing of clinical specialists, like the expert who noticed about the infection, features the situation of clinicians on the bleeding edge. In [52] the article states that man-to-man medical clinic related transmission represented 41% of the present cases in an investigation of patients at a hospital in Wuhan. We likewise realize that over 1000 emergency clinic practitioners are being affirmed contaminated. However, it is the place Infervision’s AI implementation could assist. With a CT lung check, the AI is intended to rapidly distinguish injuries of conceivable COVID-19 pneumonia, in gauging its capacity, form, and thickness, also analyze the change of numerous lung sores on the pictures, which gives all quantitative report that helps specialists to make a quick judgment. While manually reading the CT output, this may take as long as 15 minutes, whereas, AI will wrap up the picture in not more than 10 seconds. Implementation of this innovation in COVID-19 is not yet distributed into a companion inspected diary.

### A. AI Learning Techniques

A significant rebuke of clinical specialists is that the sort of media perspective of AI was the discussion had nothing to do regarding the sort of AI they were taking a shot at, which has a considerably more explicit and thin definition. They had all the earmarks of being sure about the advancement in their examination, lamenting the too-moderate interpretation from research to rehearse, regardless of whether working with the social insurance industry was viewed as an approach to quicken the interpretation. They additionally griped about the troubles prompted by enactment with regards to gathering information for examines. As indicated by them, this was the best way to see their examination financing increment and along these lines permit successful improvement of AI and an assurance of its quality. They didn’t take an interest in the conversation about the concerns in general and concentrated on their examination approach. One of the specialists in [53] employed in air transportation specialist, which talked on the human–PC communication (HCI). However, to him, even though HCI isn’t explicit to AI, scrutinizing the robotization of errands and cut-off points ought to be considered as one of the principal objectives of the mix of AI into social insurance apparatuses. He clarified that, right now, of the primary thoughts is to designate it just when it’s fundamental. Without this, it is a danger of deskilling. Deskilling is when losing the capability of the practitioner who doesn’t have the foggiest idea how to do an assignment that he did before because he quit performing it to support the machine. The related AI classified learning techniques will be described below and more emphasis will be laid upon it in later sections. In table 3, we will show the comparable differences.

**TABLE 3:** Differences Between the AI Learning Techniques

	Supervised learning	Unsupervised learning	Reinforcement learning
Definition	It learns by utilizing labeled data.	It is trained on unlabeled data.	The practitioner interacts with its environments, were as performing actions and leaning from the rewards or errors.
Problem type	Classification and regression.	Clustering and association.	Based on rewards.
Data type	Labeled data.	Unlabeled data.	Absent of predefined data.
Training	External supervision.	No supervision.	No supervision.
Approach	Inputs being labeled are mapped to the known output.	Patterns are being understood and output is discovered.	Trial and error are being implemented.
Operation	ML.	ML.	ML.
Exploration	No exploration.	No exploration.	Adapts through exploration.
Strategy	Learning algorithm and data-dependent.	Classification and data-dependent.	Learns from experience.

#### 1) Supervised Learning

**FIGURE 2.**
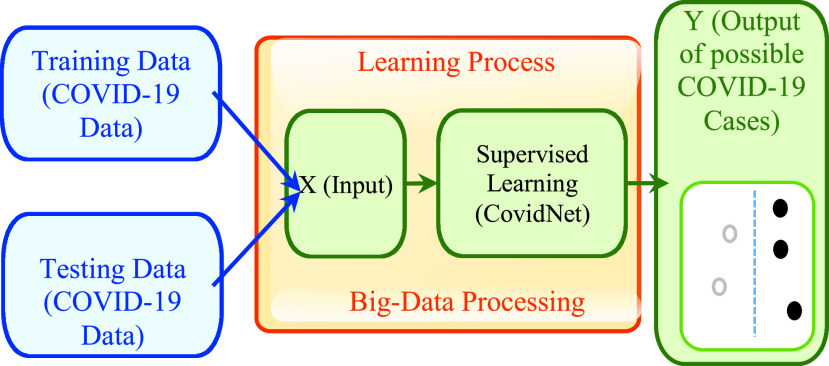
Supervised learning technique approach.

This is one of the most utilized procedures in the health system and it is commonly entrenched. This learning method utilizes information in making precise desires and learns the mapping between the yield and its relating input while gathering analysis through the learning strategy in recognizing things reliant on near characteristics. Various ways currently used in envisioning an outcome or the future outcome or gathering to a great deal of needed states [54]. The ordinary procedures finished accurately currently is the SVM, NN, and RC. In setting up these estimations, generally, the limit is being described in the medical system. It can induce the best in association between the yield and data. By then, the cost work tells the practitioner how far it can be from the accurate output, so this goes about as a data signal. The model had the choice to arrange medical data with an exactness of up to 90%. All things considered with significant learning; it has become a jump forward framework right now in all locales. Supervised learning can assemble an astute stage for programmed observing and forecasting of COVID-19. A neural system can likewise be created to extricate the visual highlights of this ailment, and this would help in legitimate checking and treatment of the influenced people. It can give the most recent updates of the patients and answer associated queries with COVID-19. The learning approach can encourage the preparation of systems by considering the contribution layer as in [55]. An Xception, depthwise based CNN approach concerning convolution distinguishable layers was proposed in [56]. It begins with two convolution layers, trailed by depthwise divisible convolution layers, four convolution layers, and a completely associated layer. Whereas in [57] it was used in classifying the bed posture utilizing various bed pressure sensors. With its capability and high-efficiency outcome, it can serve as a useful tool in combating the COVID-19. In figure 2, we illustrate the learning method, where we denote the training attribute and the testing attributes received from the patient with the symbol X, and the outcome of interest by Y. The program is fed by a preparation dataset with a label (X) which relatively yields a value (Y), and hence, the functionality will be determined according to this dataset. This functionality will be at that time utilized for arranging new information to compare the yielded values, with the suspicion that the new information complies with.

#### 2) Unsupervised Learning

**FIGURE 3.**
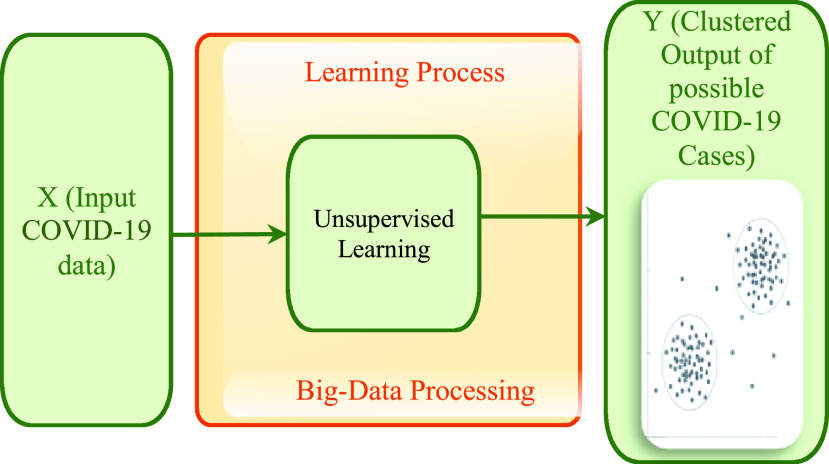
Illustration of the unsupervised learning technique.

Rather than the above learning technique that uses checked data, this learning technique uses no information signal and no names. This technique is commonly used in finding covered structures of given data and breaks it into near gatherings. It is mainly used for elucidating exhibiting and design distinguishing proof. This is a promising kind of estimation to achieve the general AI need, yet it is absent behind not in the slightest degree like the above-discussed learning technique. K-means and also the autoencoder is the most regularly known unsupervised technique. One of the most broadly perceived jobs of this learning technique in the medical line is the quirk acknowledgment [58]. The data made from the affiliation will start from equivalent scattering, if there is any sort of interference or any counterfeit read from the affiliation data, this data will be hailed an exemption and can without a lot of a stretch be hailed or seen. The K-means is a common and generally utilized grouping mechanism in image processing [59]. This calculation requires the client to indicate the quantity of cluster k to be produced. Like in [59] it was used to isolate clamor from PD flags paying little attention to conceivable few sub-classifications inside the PD and commotion gatherings. Thus, with this idea, it can be applied to CT scan images and other medical applications as well including the COVID-19. In [60] the author put forth a novel structure for this learning model for opportunistic cameras that captures moving data from the stream. Afterward, the neural system is utilized to foresee the movement of the event streamed. This movement is utilized to endeavor and to evacuate any movement obscured in the streamed pictures. In figure 3, to elaborate more on this idea, we illustrate the applied learning method, where we denote the training attribute and the testing attributes obtained from the patient by the symbol X. In this particular scenario accuracy is not gained, the goal of the approach is mainly to reveal any fascinating samples that can be discovered in the given information. And extra added/utilized information can assist in affirming or disconfirming these samples that it finds.

#### 3) Reinforcement Learning

**FIGURE 4.**
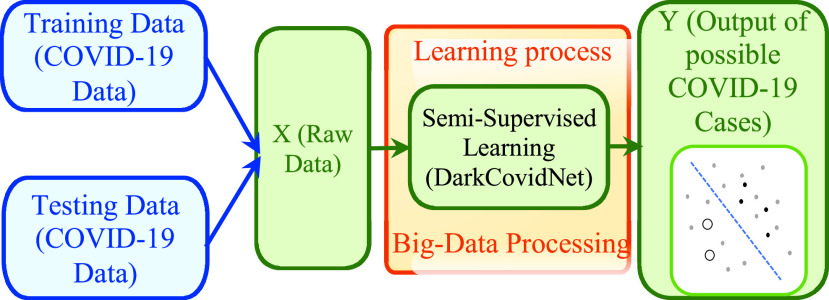
Illustration of the reinforcement learning technique.

The procedure stated is held to an utmost degree, it wears an appearance as to how individuals investigate and learn through their ordinary calendar assignments. This learning technique is neither of the two stated above; it is, considerably progressively, a kind of a crossbreed approach. In this technique, there is an alternate or single authority that makes sense of acceptable behavior in a circumstance with the end goal that they endeavor to increase their immense total prize or score. At particular advances, where the current state is given, the customer picks the best action on the explanation of a methodology, with this action the state of nature will change, and the practitioners assembles a prize sign in a brief moment in the best case, or state later, in which this is an issue referred to as delayed reward, which makes the practitioner to not choose the movement taken in the past that made way to this prize [61]. The practitioner learns an optima approach, which is the mapping from the state to move, without going before data on nature. The game plan is discovered only by performing clinical experimentation, or misuse and critical examination. This model has the capacity to decide while utilizing cooperative involvements and evaluative inputs. Unlike the customary supervised learning strategies that generally depends on one-shot, thorough and supervised signals [62]. It handles successive dynamic issues with inspective, evaluative, and deferred inputs. Such particular highlights make the RL method a reasonable option for the growing ground-breaking arrangements in an assortment of the human medical areas, where diagnosing choices or treatment regimens are generally described by a delayed and successive strategy [63]. This model empowers a specialist to learn viable procedures in consecutive dynamic issues by experimental communications with its conditions [64]. For example, the Markov Decision Process (MDP) has been utilized in the exploration of hypothetical dynamic in stochastic settings and has been utilized as a general structure to formalize the models’ issue. MDP can be implemented in different structures relying upon what data can be indicated from the earlier stage. In figure 4, we illustrate the assumed learning method, where we denote the training attribute and the testing attributes obtained from the patient with the symbol X, and the outcome of interest by the symbol Y. Here, we need an accurate prediction. However, the obtained information is not matching with the ideal yields. Thus, the assumed mechanism needs to be boosted by picking the most accurate moves to make.

## III. Deep Learning in COVID-19

The utilization of deep learning can be valuable to perceive, analyse, anticipate, and clarify the COVID-19 infection, and help in keeping up financial effects. Since the flare-up of the pandemic, there has been a scramble to utilize and investigate AI, and other information-analytical instruments, for these reasons [65]. It intends to draw speedy take-away from a quick extending conversation and develop a collection of huge data repository for research, medical arrangements, and clinical examinations. The expense of the COVID-19 pandemic, in addition to lives and financial harm, will be awful if deep learning isn’t actualized sooner or later. Improving AI, one of the most encouraging information-analytical tools, to help diminish these vulnerabilities, is of utmost interest nowadays. Encouragingly, information researchers have responded to the call. However, AI has not yet been effective against COVID-19. The utilization of AI is hampered by the absence of information, and by a lot of the uproarious and anomaly information. Defeating these imperatives requires a cautious harmony between information security and the general clinical concerns and increasingly thorough human-AI communication. It is impossible that these issues are tended to be of much assistance during the current pandemic. Rather, deep learning may help with the pandemic. Gathering analytic information on who is irresistible can be fundamental to spare lives and restricting the monetary ruin because of regulations. Authors in [65] recommended utilizing deep learning to follow and to foresee how the COVID-19 ailment can spread after some time and over space. For example, following a past pandemic, like the zika-infection, a powerful neural system was created to anticipate its spread. Models, for example, should be re-prepared utilizing information from the COVID-19 pandemic. This is by all accounts happening now. At different establishments, they proposed the utilization of calculations prepared to anticipate the occasional flue, which are presently be re-prepared on new information from COVID-19. Authors in [66] say that the deep learning framework can rapidly and dependably name chest X-ray from COVID-19 patients by classifying pneumonia as irregular, which could help accelerate analysis amid a progressing pandemic. Notwithstanding, these learning frameworks have classified the ailment accurately 87% of the time contrasted with human services experts. Such frameworks additionally are accurate 92% of the time, instead of 90% like the human specialists. Authors in [66] propose a convenient analysis and triaging for PUIs that can be critical for the control of rising irresistible viruses such as the current COVID-19. Because of the restriction of nucleic corrosive-based research, there is a pressing need to search for quick elective techniques that can be utilized by cutting edge human services personals for rapidly and precisely diagnosing the infection. In the current investigation, the authors were able to build up a deep learning program by dissecting agent CT pictures while utilizing a deep learning-based strategy. In the following, we summarize the application areas where deep learning can contribute to combating COVID-19:

• Early alerts and warning against COVID-19.
• Early COVID-19 prediction and tracking.
• Early prognosis and diagnosis.
• Social control.
• Early cure and treatments.

In the medical image analysis, deep learning algorithms can categorize, identify, and list the characteristics of diseases from medical images. It also allows the extension of theoretical targets and creates predictive treatment mechanisms for patients. It employs the deep algorithms of AI with big data. In terms of clinical operations, the complete use of these Big Data technologies would significantly reduce the experienced medical expenses. Applying a deep learning algorithm such as the Convolutional Neural Network (CNN) in diagnosis can help the specialists in setting the best treatment plan. Authors in [66] released their findings for the detection of strabismus by using CNN. A CNN model has been trained based on a collection of data gathered by an eye motion tracker, including visual data from both normal and strabismus visions while considering gazing deviation (GaDe). After training with several GaDe images, the CNN model was successful in identifying strabismus. The emerging Internet of Medical Things (IoMT) paradigm, which relies on intelligent medical systems, can also track significant human health issues continuously. In this paradigm, robots are providing help to elderly and disabled people via their smart homes and virtual assistants. Epidemic diseases can be also tracked and avoided by integrating the data collected with the IoMT sensors and the information obtained from the associated health system [66].

### A. Deep Learning Difficulties

This model can assume a fundamental role in relieving the effect of the COVID-19 pandemic. Moreover, at present, deep learning is still in the prefatory stages. Difficulties and impediments towards the utilization of deep learning in fighting the COVID-19 can be listed as follows:

1) In yielding a dependable and precise outcome, the deep learning model requires a generous measure of the training information. In any case, inferable from the remarkable idea of the pandemic, there is a shortage of recorded information on which to train a deep learning model, which has subsequently rendered the model wasteful [67].
2) It isn’t just the nonattendance of open information that has blocked the exhibition of the deep learning model, an excessive amount of irregular and anomaly information has additionally introduced a test to the productive utilization of the deep learning advancements. The Trends’ of Google Flu bombed activity reveals insight into the way that a downpour of information hubris can immerse deep learning algorithms and restrain their working [67].
3) Another restricting challenge, is the intrinsic suspicion that every single imaginable possibility in some random circumstances are equivalent to the ones showed in the dataset they have been prepared on [67].
4) The utilization of deep learning for mass observation has been also seen by numerous individuals as a violation of protection. Although at present, individuals have caught the way that general medical concerns are a higher priority than information security concerns, the protection traps related with the utilization of deep learning have ingrained a feeling of dread among the public that administrations may keep observing them significantly after the pandemic is over [68].
5) Another restriction of deep learning in its present structure is its reliance on human information. Human skill is principal to control the execution of the deep learning methods and have an effective influence on the fight against the COVID-19 pandemic [68].

Regardless of the above-mentioned difficulties, the deep learning frameworks commitment to the battle against the COVID-19 pandemic can’t be ignored. As of late, deep learning innovation has made shocking advances in the medical field. Such improvements serve to demonstrate the capability of deep learning in helping the COVID-19 pandemic administration sector.

## IV. Commuting AI Techniques in Combating COVID-19

Simulation preparation has developed exponentially around the globe in recent years. This is in huge part connected with the ordered residency preparing arrangement for doctors which started since the year 2015. In any case, the significant focal point of those re-enactments was to meet the instructive needs of various preparing programs or to enhance certain educational programs [69]. In the wake of recognizing the requirement for preparing, recreation has become an incredible weapon battling against the virus, as it can guarantee quiet security as well as giving a sheltered learning and preparing condition for HCWs to manage the COVID-19 [70]. In [71] Boltzmann function-based regression analysis was proposed in simulating and forecasting the SARS-CoV-2 virus. This method was applied to the cumulative confirmed cases in the province of Wuhan. Boltzmann function gives a direct estimate of potential confirmed cases. In addition to the Boltzmann function, it doesn’t require much more detailed data for analysis. In [72] [73], the cumulative cases are only needed. Also, it allowed the forecasting of future courses in other cities. However, the estimates of the results are not guaranteed due to uncertainty in the data being reported.

However, machine learning techniques can be applied to provide an accurate predicting method with the provided data set. Pattern recognition can also be suggested in recognizing similarities in other infected hosts. In [74] the virus is similar to the SARS virus due to an intermediary host from bat to humans. The civic cat was said to be an intermediary host between bat and man. While the SARS-CoV-2 virus can highly be related to that of SARS, with a high percentage of the bat sequence, pattern matching can be carried out in studying the sequences of animals situated in the seafood market of origin. This is a benchmark for us. Not long before the New Year, we took a rollcall at what number of individuals left Wuhan longer than a day, and this data originates from clinical information. The clinical research gives information on movement around Wuhan, these clarify the usage of AI models which utilizes this information to anticipate the most probable area of where novel coronavirus may show up straightaway and this may illuminate where and how to run fringe checks. This additionally vouches for the quality of observing news reports and web-based life to help recreate the movement of an episode and to give point by point quiet level information with regards to the health crisis. In the subsections below we will discuss this individual implementable technique. In table IV we discuss the difference between the applications of the techniques.

**TABLE 4:** Differences Between the Above Methods

Areas	SVM	Neural networks	Deep learning	Natural language
Pre-processing phase	Need	Need	Doesn’t need	Need
Data set size	Small	Large	Large	-
Training time	Short	Short	Long	Short
Hardware requirement	Simple	Simple	High end	-
Accuracy	Lesser	High	High	High
Training time	Lesser	Medium	Longer	-
Interpretability	Easy	Easy	Difficult	Easy

### A. Support Vector Machine

**FIGURE 5.**
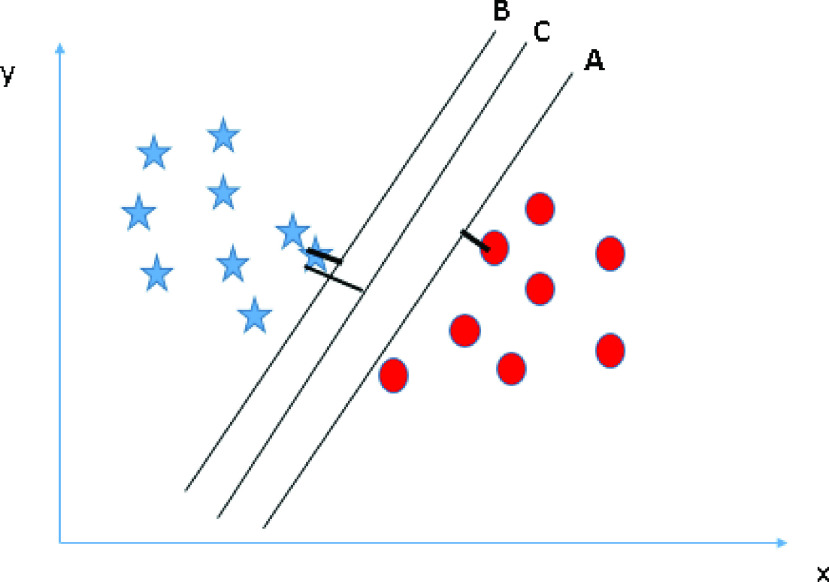
SVM illustration.

This strategy is predominantly utilized for arranging the subject into two states or groups, whereas the result Y is regarded as a classifier: were Y = 1 or −1 speaks to if the nth patient is in the first or second state, separately. This technique is stretched out for situations with multiple states. The fundamental supposition that will be, that the subjects can be isolated into two gatherings through a choice limit which is characterized on the trait X, The preparation objective is to locate the ideal weight so the subsequent arrangements that concur with the results however much as could reasonably be expected, that is, with the littlest misclassification blunder, the mistake of ordering a patient into an inappropriate state. Instinctively, the best loads must permit a) The indication of $\text{a}_{\mathrm {i}}$ to be equivalent to Y therefore the grouping is right, and (b) $\text{a}_{\mathrm {i}}$ most be distant away from 0 so the vagueness characterization will be limited. Were $\text{a}_{\mathrm {i}}$ is gotten by a choice standard which is if $\text{a}_{\mathrm {i}} >0$, the nth patient is arranged to state 1. These can be accomplished by choosing loads that limit a quadratic misfortune work [75]. Besides, accepting that the new patients originate from a similar populace, the subsequent loads may be implemented to arrange these latest patients dependent on the patient’s characteristics. Figure 5 illustrates the SVM method. Here, we present the hyper-plan A, B, and C, and all are isolating the classes well. Presently, that hyper-plan can be distinguished effectively. By expanding the separations between the closest information point and the hyper-plan, it will assist in choosing the privilege plan. The margin is referred to as the distance between them. Another lightning explanation behind choosing the hyper-plan with a higher edge is the robustness (e.g., the hyper-plan C). On the off chance that we select a hyper-plan having a low edge, at that point, there will be a high possibility of miss-characterization. A significant property of this method is that it gives the assurance of the used model parameters, and thus the arrangement is constantly ideal. Besides, many existing enhancement instruments are promptly appropriate for the execution of this method. In that capacity, this method has been widely utilized in clinical research. For example, in [76] it was applied to distinguish imaging biomarkers of neurological and mental sickness. In [77] it was evaluated to be utilized in the determination of malignant growth. In [78] it was utilized to mix this method with other measurable devices to accomplish early discovery of Alzheimer’s sickness. In [79] it was utilized in testing the intensity of a disconnected machine or man’s interface which controls the upper appendage prostheses.

### B. Neural Network

With this technique, it very well as an augmentation of direct relapse to catch complicated non-linear connections admits input factors and a result. In the neural system, the relationship admits the result, and also the info factors are delineated by various shrouded layers with a mix of pre-indicated usefulness. The objective is to appraise the loads through information and result information with the goal that the normal blunder between the result and their forecasts is limited. We depict the technique in the accompanying model. In [80] it was utilized in stroke finding. In their examination, the info factors Xi$\ldots$ Xp is given by p=16 with stroke-related manifestations, which includes paranesthesia of either the leg or hand, intense disarray, sight, issues with portability, and so on. The output Y is twofold: Y=1/0 demonstrates the nth patient is not diagnosed with a stroke. However, the yield criteria of intrigue are in the likelihood of the cause of stroke that is $\text{a}_{\mathrm {i}}$. Moreover, the preparation objective is to discover the load’s weight, which limits the expectation blunder. The minimization can be performed through standard improvement calculations, for example, neighborhood quadratic estimation or slope plummet streamlining that is remembered for both MATLAB and R. If the new information originates from a similar slope plunge advancement, that is remembered for both MATLAB and R. If the new information originates from a similar populace, the subsequent weight can be utilized to anticipate the results dependent on their particular characteristics.

**FIGURE 6.**
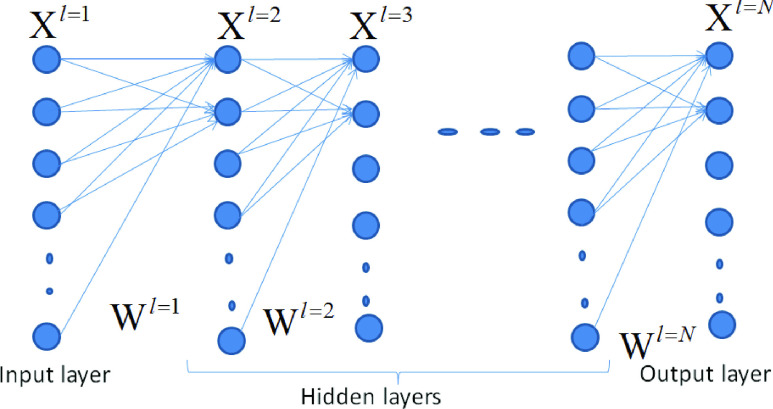
Neural network illustration.

Comparative strategies have been utilized to analyze malignancy like in [81], where the sources of information are the PCs assessed from 6567 qualities and the results are the tumor classifications. In [82] it was utilized to anticipate bosom malignant growth, with the sources of info being the surface data from mammographic pictures and the results being tumor markers. While in [83] it was utilized a progressively modern model of the neural system to analyze Parkinson’s sickness dependent by the contributions of motor, also non-motor problems and neuroimages. Figure 6 gives an illustration of the neural network and its utilized hidden layers, the X’s are inputs, and W’s are weights per neuron. The output Y is calculated based on utility function $f$ as follows: $y=f\left ({u }\right)$ with$u=\sum \nolimits _{i=1}^{i=I} {\left [{ w\left ({i }\right)x\left ({i }\right) }\right]+b}$, where $b$ is the bias, and $u$ represents the internal signals between the neurons. This model is roused by the human cerebrum and comprises of different associated neurons. The system comprises of a layer of information neurons that is the input neuron, and a layer of yield neurons that is the output neuron and various alleged shrouded layers in the middle, known as the hidden layer.

### C. Deep Learning

This strategy is an advanced augmentation of the old-style neural system method. One can see deep learning as a neural system with numerous layers. Quick improvement of present-day computing gives rise to deep learning in figuring out how to develop neural systems with an enormous number of segmented layers that can be infeasible for old-style neural systems. In that capacity, this strategy investigates progressively complex non-straight examples in the information. Another explanation behind the ongoing prominence of the technique is because of the expansion of the volume and multifaceted nature of information [84]. What’s more, a larger part of this method is implemented in images investigation, which bodes well with the insight that pictures are usually mind-boggling and huge capacity. Unique concerning the old-style neural system, this learning makes use of increasingly concealed layers therefor the estimations may manage complicated data consisting of various structures [85]. Utilizing it in clinical implementation, the generally applied deep learning calculations implement CNN known as convolution neural network, also neural intermittent system, also the deep neural system, and lastly deep convection system. This model is created in the survey of the inadequacy of the traditional AI calculations when taking care of huge dimensional information, which is, information containing an enormous amount in qualities. Customarily, AI calculations are intended to investigate information when the quantity of qualities is little. Be that as it may, the picture information is normally high-dimensional because each picture ordinarily contains a great many pixels which are the characteristics. One of the arrangements is in performing measurement decrease: first, make a preselection of the subset of a pixel as highlights, secondly, afterward, play out the AI calculations with the subsequent lower-dimensional highlights. In any case, heuristic element choice methods may lose data in the pictures.

The CNN model was initially put forth and supported for a high-dimensional picture investigation by creators in [86]. The contributions for the model are the standardized appropriate pixel esteems regarding the image. This model at this point moves the esteems pixel regarding the images by weighting with the convolution segments also inspecting the subsampling segments then again. Finally, the yield will be recursive capacity by the weighted information esteems. However, the loads are prepared in limiting the normal mistake amidst results and expectations. The utilization of this model has been remembered for well-known programming bundles [87] and TensorFlow [88]. As of late, the model with high regard is effectively executed in the clinical zone in assisting ailment finding. In [89] it was utilized to analyze inherent waterfall ailment by learning visual pictures. The model outputs over 91% exactness in findings and proposing treatments. Whereas, in [90] it was utilized to recognize skin disease from clinical pictures. The extents of effectively anticipated threatening injuries and kind-hearted sores are both over 90%, which shows the prevalent presentation of the model. In [91] it was applied to distinguish diabetic referable retinopathy by a huge volume of fundus retinal photos. In affectability, also particularity in the calculation is similarly more than 90%, this shows the viability of utilizing this system on diabetes determination. It merits referencing is that every one of these implementations, the exhibition of this model is serious over experienced doctors regarding the exactness for ordering both typical and malady situations. The utilization of AI for COVID-19 pandemic has been on heavy demand in recent times. This innovation assumes a significant job to identify a group of cases and to anticipate where this infection can influence in the future by gathering and dissecting every single past information. Medical associations are in a dire requirement for dynamic advancements to deal with this infection and help them in getting appropriate recommendations progressively to maintain a strategic distance from its spread. AI is capable to emulate human insights. It can likewise assume an imperative job in comprehending and recommending the advancement of an antibody for COVID-19 [92]. Computed Tomography (CT) has up to this point become a quick strategy to determine patients who have COVID-19. In any case, the presentation of radiologists in the conclusion of COVID-19 was moderate. In alike manner, extra examinations are expected to improve the presentation in diagnosing COVID-19. Albeit a research center test is the current apparatus, it consumes time, and it is forcing significant expenses and requires a well-prepared lab for examination [93]. The subsections below overview the associated AI methods which helps in overcoming the aforementioned challenges.

#### 1) Single Deep Learning Methods

In [94] authors utilized a google deep learning tool called inception v3 for the classification of skin injury, having in mind utilizing these procedures for other possible diseases. The deep learning model was trained by utilizing about 129,450 clinical pictures by the authors to perceive noncancerous skin growth called seborrheic keratosis, keratinocyte, and carcinomas basal cells, nevi tumors, and life-threatening malignant tumor. Authors in [95] convey a different type of method for the classification of the proceedings of the COVID-19. They put together their method concerning a deep learning fine-tuned ResNet-152 model. The made method permits the classification of pneumonia and other stated conditions as above. However, in [73] authors put forth a deep learning method for detecting the 2019-ncov outbreak by utilizing ResNet-101 and Inception-v4. The authors perceived various traits of pneumonia in their findings. They utilized a few outer datasets for making deep learning models. Authors in [96] also utilized google tools for characterizing skin sore by using convolutional neural method. To begin with, this strategy assembled harmful and non-harmful sorts of skin injuries. Secondly, the technique further partitioned threatening and non-threatening sorts into epithelial and melanocytic states. In like manner, they further isolated each gathering into more subcategories. Subsequently, they could perceive skin injuries of up to twenty-one classes. While in [97] with the help of the above-mentioned technique the authors utilized deep convolutional neural systems for classifying CT scans with tools like Inception v3, DenseNet-210, and ResNet-152. These models identify chest diseases, basal cell carcinoma, and also like the one stated above. However, the ResNet-152 models outflank different models for melanoma and melanocytic nevi identification. The Inception v3 model beats different models for actinic keratosis and favourable CT scan arrangement. DenseNet-210 gave the best outcomes to basal cell carcinoma and dermatofibroma arrangement. The majority of these tools have been utilized in combating the COVID-19 but it is still not effective because of the lack of the large dataset.

#### 2) Ensemble Deep Learning Methods

In [96] authors utilized a gathering of convolutional neural system tools called ResNet for perceiving fever-like cough for the COVID-19. This methodology was put forth concerning creating a few deep models for ResNet. This tool expands on various expansion information. They melded every one of the model probabilities in distinguishing proof for melanoma and seborrheic keratosis classes for skin class. Authors in [99] utilized the AlexNet tool and google tools for convolutional neural systems and joint model yield probabilities for melanoma order, this can also be proposed for fluorescence polymerase chain reaction for the COVID-19. Four distinct strategies lead to the last probabilistic polymerase chain forecast. The combination systems are the maximal probabilities, product, sum and the larger part casting a ballot. This model outflanks single deep learning strategies like AlexNet, GoogLeNet, and VGGNet. The authors in [100] utilized convolutional neural systems for classifying skin sore with tools like DenseNet, ResNet, and Inception V3. They anticipated skin injuries utilizing every one of these neural models, and again assessed sore sorts by combining yield evaluations of every one of the models. The examination presents two preparing systems. One of them is to prepare the models utilizing unique pictures. Another is to prepare the models utilizing edited skin pictures around the locale of intrigue. However, in [101] the authors produced deep learning models and combined these models for forecasting skin injury with tools like DenseNet, Inception V3, and ResNet. The creator’s combination approach prompted higher exactness of forecast than single neural network models. In [102], for chest computed tomography location, a joint two ResNet models were utilized with the above method in mind. The creators put together their methodology concerning convolving pictures utilizing a 3-dimensional model and afterward consolidating saliency maps of the two models. Finally, they assessed the chest images utilizing joint yield saliency maps. Just like in [103] were the authors perceive melanoma among seborrheic keratosis and nevus they utilized tools like two ResNet models. In the first place, the creators utilizing every one of the ResNet models by anticipating melanoma. The presentation results have indicated that the two ResNet models outflanks every one of the single ResNet models.

#### 3) Feature-Based Deep Learning Methods

In [104] the authors include the extraction and afterward classifying these traits utilizing bolster vector machines with tools like AlexNet, and VGGNet. The creators in like manner combined separated highlights for skin sore acknowledgment. This examination shows that melded highlights for sore expectations beat the qualities of every one of the deep learning models. In [105] it was utilized with various component pictures for recognizing CT imaging features of the novel coronavirus. The outcomes have indicated that intertwining handcraft and deep highlights gave higher precision in the images. In [106], in figuring out how to perceive dangerous melanoma and seborrheic keratosis skin injuries the authors proposed a deep learning based on Gabor wavelet. The technique represented the skin picture into seven directional Gabor band and afterward displayed every one of the portrayals utilizing the deep learning tools like AlexNet, In [105] The examination indicated that COVID-19 commonly shows on CT with respective ground-glass and consolidative aspiratory opacities. Nodular opacities, insane clearing design, and a fringe dissemination of illness might be an extra highlight in early conclusion. The analysts likewise noticed that lung cavitation, discrete pneumonic knobs, pleural emissions, and lymphadenopathy are typically missing in instances of 2019-nCoV.

### D. Natural Language Processes

Pictures, hereditary information, and EP are machine-justifiable with the goal that the AI calculations can be legitimately performed after appropriate pre-handling or quality control forms. Notwithstanding, huge extents of clinical data are story content just like, physical assessment, reports from the clinical lab, useful notes, and also release outlines that are not structured and immense for PC programs. With this unique situation, this method focuses on removing valuable data from the story content which helps medical dynamics [107]. The methods pipeline consists of these principal parts: (a) preparing content and (b) order. Through content preparation, the NLP recognizes a progression of malady applicable catchphrases from the medical notes dependent on its verifiable databases. At that point, the subsets catchphrases are chosen by analyzing their consequences for the order of the typical and unusual scenarios. The approved catchphrases at that point enter and advance the organized information to help clinical situations. This pipeline was created to help the clinical situation on cautioning medical care courses of action, checking antagonistic impacts, etc. For instance, [108] indicated that the presentation of this method for utilizing the reports from a chest X-beam can assist the said anti-toxin relative framework in alarming practitioners for conceivable needs for hostile to infective treatment. Authors in [109] utilized this method in screening the research center based on unfavorable impacts. Besides, the pipelines can assist in sickness analysis. Just like, [110] distinguished 14 cerebral aneurysms sickness-related factors through executing this method based on medical notes. However, the subsequent factors are effectively utilized for characterizing the cerebral sickening patient and the normal patient, given with 85% and 96% precision values by the preparation and approval tests, individually. Creators in [111] executed the NLP to remove the fringe blood vessel malady related watchwords from story medical notes. Thus, the watchwords, afterward are utilized to order the typical and also patients with fringe blood vessel ailment, which accomplishes over 91% accuracy.

## V. Datasets

The COVID-19 has prodded enthusiasm for large information to follow the spread of the quick-moving pathogen and to design ailment anticipation endeavors. Yet, the critical need to contain the episode shouldn’t cloud pondering huge information’s capability to accomplish more damage than anything else.

Organizations and governments overall are tapping the area information of a large number of web and cell phone clients for intimations about how the infection spreads and whether social removing measures are working. Dissimilar to reconnaissance quantifies that track the developments of specific people, these endeavors break down huge informational collections to reveal designs in individuals’ developments and conduct through the span of the pandemic. Specialists and practitioners are progressively utilizing man-made brainpower, AI, and characteristic language preparing to follow and contain coronavirus, just as addition an increasingly exhaustive comprehension of the illness. In the months since COVID-19 hit the world, analysts have been working diligently attempting to reveal the idea of the infection, like why it influences some more than others, what measures can help diminish the spread, and where the ailment will probably go straightaway as well as the likes of skin lesions. Scientists are utilizing Big Data and examination to all the more likely grasp coronavirus from various edges. The foundation as of late reported that it would offer government substances, look into associations, and industrial access to inventive AI apparatuses, just as specialists in information and general wellbeing help to battle COVID-19 [112].

No nation knows the complete number of individuals contaminated with COVID-19. The known fact is the contamination status of the individuals who have been tried. Each one of the individuals who have a lab-affirmed disease is considered affirmed cases. This implies the tallies of affirmed cases rely upon how much a nation test. Without testing, there is no information. Testing is our window onto the pandemic and how it is spreading. Without information on who is contaminated by the infection, we have no chance to get of understanding the pandemic. Without this information, we cannot know which nations are progressing nicely, and which are simply underreporting cases and passing. To decipher any information on affirmed cases we have to know how much testing for COVID-19 the nation does. Like the author in [113] was able to get a total of 618 transverse-section CT samples which includes 219 from 110 patients with COVID-19 as the dataset of the infected hasn’t being released to the public. Table 5 shows the accessible datasets in writing so far related to their references concerning other viruses.

**TABLE 5:** Relevant Dataset

Dataset	COVID-19	Head CT	Basal cell carcinoma	Melanoma
2020 ARXIV [112]	618	-	-	-
2019 ISIC [113]	-		3323	4522
2018 ISIC [114]	-	14851	-	-
2017 ISIC [115]	-	-	-	521

### A. Battling COVID-19 With a Comprehensive Dataset

Big information has been demonstrated with the ability to help battling irresistible illnesses like COVID-19 [116], [117]. Huge information conceivably gives various promising answers for helping the battle against COVID-19 pandemic. By consolidating with AI investigation, the dataset encourages us to comprehend the COVID-19 as far as flare-up following, infection structure, sickness treatment, and immunization fabricating [118], [119]. For instance, enormous information related to advance AI-based devices can manufacture complex reenactment models utilizing coronavirus information streams for flare-up estimation. This would help medical offices in observing the coronavirus spread and getting ready for better preventive estimations [119], [120]. Models from huge information likewise underpins future expectation of COVID-19 pandemic by its information accumulation capacity to use a lot of information for early identification. Also, the dataset examination from an assortment of certifiable sources including tainted patients can help execute enormous scope for COVID-19 examinations to create far-reaching treatment arrangements with high dependability [122], [123]. This would likewise help social insurance suppliers to comprehend the infection improvement for better reaction to the different treatment and conclusions.

### B. Datasets and the Spread of COVID-19

Another task of the dataset is to follow the COVID-19 spread, which is of principal significance for human services associations and governments in controlling effectively the coronavirus pandemic [124]. Various recent rising arrangements utilizing large information have been proposed to help follow up the COVID-19 spread. For example, the examination in [125] recommended a major information-based system for following the COVID-19 spread. A numerous straight model is fabricated utilizing neighborhood populace and air travelers as evaluated factors that are helpful to measure the fluctuation of revealed cases in China urban areas. to be more explicit, the creators utilized a Spearman relationship examination for the day by day client traffic from Wuhan and the all-out client traffic in this period with the quantity of 49 affirmed cases. The explanatory outcomes show a high relationship between the positive disease cases and the populace size. The creators in [126] thought about utilizing both enormous information for spatial investigation strategies and Geographic Information Systems (GIS) innovation which would encourage information obtaining and the joining of heterogeneous information from medical information assets, for example, governments, patients, clinical labs and the general population.

## VI. Performance Evaluation

In deep learning, performance assessment is a basic task. With this, with regards to CNN models, we can depend on an AUC - ROC Curve. At the point when we have to check or imagine the exhibition of the multiclass order issue, we utilize AUC and ROC bend. It is one of the most significant assessment measurements for checking any characterization model’s presentation. The ROC is a likelihood bend and AUC represent the degree or proportion of detachability. It tells how much model is fit for recognizing classes. The higher the AUC curve, the better the model is at anticipating 0s as 0s and 1s as 1s. Similarly, the higher the AUC, the better the model is at recognizing patients with illness and no infection. The framework proposed by [127] utilizing Riesz and Gabor changes acquired a 98.73% accuracy in distinguishing lunge illness. It expanded to 99.53% accuracy with the mix of Riesz, Gabor, fractal measurement, dark level co-event grid, and dim level run-length framework-based highlights. Authors in [128] demonstrated that a deep CNN could distinguish lung knobs with an opposition execution metric of 0.7967. They also put forth a thickness based spatial bunching with clamor DBSCAN calculation to improve the location affectability of the system. Likewise, the CAD framework dependent on a 3D skeletonization was proposed by [129]. This can help radiologists to separate lung knobs from interferential vessels. Changing a picture to the time recurrence area by the optimal fractional S-transform (OFrST) strategy can give important data and highlights, which can be utilized to analyze ailments. This technique was applied to lung CT pictures by [130] to identify and separate knobs from the vessels. In [131] they utilized the Teager–Kaiser vitality (TKE) in the time–recurrence area to get the vitality circulation and describe lung knobs with a sensitivity of 97.87%. The author reasoned that CNN was better than a deep belief network (DBN) and stacked denoising autoencoder (SDAE) in diagnosing threatening lung knobs with an AUC of 0.899. Moreover, CAD frameworks are a valuable device to screen lung work after transplantation. With this, a framework dependent on deep learning on how to arrange COVID-19 contamination versus another typical and viral pneumonia sickness can be proposed. An estimated hypothesized deep learning would help radiologists in diagnosing contamination identified with COVID-19. A convolutional neural system (CNN) to analyze diseases identified with the COVID-19 end up being proficient. The zone under the collector working trademark (ROC) bend (AUC), exactness (ACC), affectability (SE), and explicitness (SP) execution merits are utilized to test the precision of the strategies [132].

### A. Accuracy

The accuracy of a strategy decides how right the qualities are anticipated. The score utilizes a blend of accuracy and review to compute a fair normal outcome. Eq. (1) tells the best way to compute this exactness, where TP, TN, FP, and FN are genuinely positive, genuine negative, bogus positive, and bogus negative individually. In [132] the creators show the exactness level of up to 90% when contrasting CT examination with that of the COVID-19 patients.

\begin{equation*} Accuracy=\frac {\mathrm {TP+TN}}{\mathrm {TP+TN+FP+FN}}\tag{1}\end{equation*}

Among all systems, the best performance was accomplished by ResNet-101 and Xception. ResNet-101 could recognize COVID-19 from non-COVID-19 cases with an AUC of 0.994. Xception accomplished an AUC of 0.994. Be that as it may, the presentation of the radiologist was moderate with an AUC of 0.873. ResNet-101 can be considered as a high affectability model to portray and analyze COVID-19 diseases and can be utilized as an adjuvant device in radiology offices.

### B. Sensitivity

Sensitivity shows what number of the right outcomes are found. Eq. (2) tells the best way to figure this precision, where TP and FN are genuinely positive, and bogus negative separately. In any case, in [132] the affectability was at its top with a 90% on second examination, while contrasting CT filter with that of the COVID-19 patients. The deep learning models utilized have similarly demonstrated high location results with ResNet-152 design being the best of them.

\begin{equation*} Sensetivity=\frac {\mathrm {TP}}{\mathrm {TP+FN}}\tag{2}\end{equation*}

The best execution was accomplished by ResNet-101 and Xception. ResNet-101 could recognize COVID-19 from non-COVID-19 cases with a sensitivity of 100%. Xception accomplished a sensitivity of 98.04%. Be that as it may, the presentation of the radiologist was moderate with an affectability of 89.21%. ResNet-101 can be considered as a high sensitivity model to describe and analyse COVID-19 diseases and can be utilized as an adjuvant apparatus in radiology divisions.

### C. Specificity

Specificity decides the reproducibility of the estimation or what number of the forecasts are right. In order words, it gauges the extent of negatives effectively ordered. Eq. (3) tells the best way to ascertain this precision, where TN and FP are genuine negative and bogus negative individually. Notwithstanding, as opposed to the affectability the explicitness was at a top with a 91% imprint by creators in [132], while contrasting CT filter with that of the COVID-19 patients.

\begin{equation*} Specificity=\frac {\mathrm {TN}}{\mathrm {TN+FP}}\tag{3}\end{equation*}

It was understood that ResNet-101 and Xception were the best. ResNet-101 could recognize COVID-19 from non-COVID-19 cases with a specificity of 99.02%. Xception accomplished a specificity of 100%. Be that as it may, the exhibition of the radiologist was moderate with an explicitness of 83.33%. ResNet-101 can be considered as a high specificity model to portray and analyse COVID-19 contaminations and can be utilized as an adjuvant apparatus in radiology divisions.

## VII. Incorporating Cloud Computing in Combating COVID-19

The previous months have been one of segregation, both socially and genuinely. In an offer to forestall COVID-19 from spreading, our physical universes have become geofenced to envelop our home and short neighborhood strolls. Accordingly, our virtual lives have woken up in a variety of wealth as we endeavor to supplant our physical real factors with virtual devices that make a computerized change of our social associations and everyday lives. On the off chance that one innovation had the option to spark a brilliance amid office termination, isolation, social separation, and uneasiness, it’s distributed computing. Cloud administrations have empowered us to proceed with our advanced lives through different applications. Nonetheless, many cloud suppliers are helping the battle against COVID-19 in a variety of ways. As of late a business association propelled its first Salesforce Care answer for Healthcare Systems, structured explicitly for human services suppliers encountering an inundation of solicitations due to COVID-19 [133], [134]. They have additionally extended deals with extra free in helping organizations in any industry remain associated with partners, in any event, when everybody is working remotely. The consideration arrangements are accessible promptly and can be set up rapidly. A significant association has offered AI-controlled distributed computing stage and supercomputing bunch for nothing to control worldwide research foundations to quicken viral quality sequencing, protein-screening, and other research in treating or forestalling the Coronavirus.

Elite Computing Consortium has been sorted out to unite offices around the globe and different offices and scholarly pioneers to give access to the world’s most remarkable superior figuring assets on the side of COVID-19 research [135]. The consortium will deliver an exceptional measure of processing power for the research facility, which is the quickest supercomputer on the planet. Likewise, with this framework, it permits analysts to run huge quantities of computations in the study of disease transmission, bioinformatics, and atomic displaying. This makes tests conceivable that would somehow, or another take a long time to finish whenever worked on more slow, customary registering stages. Cutting edge advancements are probably going to get the most approval– particularly by the media. In any case, new businesses have been faithful clients of cloud suppliers [136]. They ought to be bolstered in their endeavors to remain above water. They may not all be as alluring as supercomputers, yet they give employments and administration their nearby economies. Their capacity to keep on supporting their client base using cloud administrations will be a crucial piece of their recuperation. We’re in phenomenal occasions all around and this is an open door for huge tech to step up and bolster their customers and lead the path in helping organizations endure. Some of the cloud services are discussed below [137].

### A. Virtual Network

As we probably are aware, a virtual system associates administrations and assets like virtual machines and database applications with one another and the remainder of the web through a safe, encoded, and private system. This module gives a structure that gives your undertaking cloud foundation substance. Virtual frameworks organization supports the association of varying organizations and devices on a single hardware called a virtual organization switch. The centralization of control lessens the cost and unusualness of working and keeping up gear and programming differentiated and controlling different separate devices in for the most part confined land territories. Upkeep staff and directors can present device drivers, perform tests, and resolve issues on the remote machines from a singular zone. It might be basic to introduce virtual systems association programming on the remote PCs or servers to abuse this progression. A few dealers offer virtual systems association programming. Two or three merchants offer wide virtual structures association associations, permitting businesses to sort out heads to redistribute work and assets for the vendor. With this, professionals can in-coordinate their thoughts for all intents and purposes with the assistance of the virtual system in fighting the COVID-19 issues [138].

The central restriction of virtual frameworks organization is the way that particular issues can be settled unmistakably by direct physical contact with the hardware being referred to. Models join broken wires, set drives, and imperfect chips. Occasionally, virtual frameworks organization may make it possible to circumvent a discomfort by abusing elective resources until an expert who can fix it or substitute it. Virtual systems administration depends on physical PC organizing standards; however, its capacities are generally programming driven. The VMs impart by tending to the predefined IP address of every goal virtual machine. Essentially, a virtual neighbourhood is made through programming based virtual switches that give a correspondence between all virtual and associated machines. Virtual systems administration additionally might be executed on virtual machines that are introduced or sent on Internet-empowered physical servers [139].

### B. Virtual Server

This is most occasions alluded to as a virtual machine, this is a type of virtualization in the cloud that mimics a physical server, and which is managed by cloud suppliers in having the equivalent server with various cloud buyers in giving cloud purchasers single mimicked virtual server. The quantity of examples a given physical server can share is restricted by its ability. As an aware system, the virtual server speaks to the most central structure square of cloud situations. Each virtual server can have various IT assets, cloud-based arrangements, and different other distributed computing systems. The launch of virtual servers from picture documents is an asset distribution process that can be finished quickly and on-request. Cloud shoppers that introduce or pay by service virtual servers may redo their environment autonomously from various cloud customers that may be making use of the virtual servers facilitated by the equivalent fundamental physical server [140].

However, a virtual server copies and gives server functionalities. In place of a completely distinctive submitted server, two or three virtual servers may be carried out on a single server. An individual virtual server is consigned to another OS, programming, and autonomous reboot provisioning. A virtual server that is for Web empowering, page administrators, or Internet authority affiliations (ISP) may have obvious space names, IP addresses, email affiliation, record lists, logs, and assessment. Also, security frameworks and passwords are kept up as though they were in a submitted server condition. Also, to diminish Web empowering costs, server programming establishment provisioning is a significant part of the time accessible. A surge of virtual servers in a physical machine may impel the resource being covered and if a virtual server uses a more noteworthy number of focal points than another, the execution it gives can take everything into account. At present, the virtual part just infers that it’s not a submitted server, that is, the entire PC can’t run the server programming. Virtual Web servers are an incredibly notable strategy for giving simplicity web encouraging organizations. As opposed to requiring an alternate PC for each server, numerous virtual servers can co-live on a comparable PC. When in doubt, execution can’t and each site goes about just as it is being served by a committed server. Regardless, if an unreasonable number of virtual servers harp on a comparative PC, or if one virtual server starts storing resources, Web pages will be passed on even more bit by bit [141].

### C. Virtual Storage

Virtual storage alludes to the virtualized type of a capacity medium, as such it exists as a build inside a virtual domain. This goes about as a deliberation between the client and the genuine stockpiling equipment. Virtual storage is a staple element of distributed computing and is accessible for the most part as online stockpiling or reinforcement. Virtual storage used to be synonymous with virtual memory, which was an augmentation of the fundamental memory gave through optional stockpiling. In any case, with the appearance of distributed computing, the term has gotten progressively exacting, just importance stockpiling that has been made in a virtual domain. A virtual stockpiling medium or gadget is typically likewise connected with virtual machines. A virtual machine appears to the client as an undeniable PC with a working framework and all the fancy odds and ends, and obviously with its very own capacity drive [142], [143].

Storage virtualization is a framework organization practice that permits decoupling the physical association of the equipment from its intelligent portrayal. Utilizing this method, clients don’t need to be stressed over the particular area of their information, which can be distinguished in utilizing a sensible way. Storage virtualization permits us to bridle a wide scope of storerooms and speak to them under a solitary legitimate record framework. There are various systems for storage virtualization, one of the most famous being system-based virtualization by methods for storage territory systems. This model utilizes a system available gadget through an enormous transfer speed association with give storerooms [144].

### D. Virtual Middleware and Application

Middleware is a product stage that sits between an application/gadget and another application/gadget. It makes the association between any two customers, servers, databases, or even applications conceivable. Cloud middleware is constantly available to the client as a remote programming stage for correspondence or the board of information. Ordinarily, it is arranged between the working framework and an application, cloud middleware gives various functionalities to the client. It helps in the formation of business applications, encourages simultaneousness, exchanges, stringing, and informing and offers an assistance part engineering structure for making administration situated design applications. Web servers, application servers, and databases are instances of cloud middleware. Middleware programs give correspondence administrations and fill the need of a detachment with the goal that various applications can send and get messages. Various applications arranged at various physical areas can be tied together to play out an undertaking through cloud middleware [145], [32].

## VIII. Conclusion

Typically, the artificial intelligence tool is arriving at the clinical field in present times. It is presently a reality that we should face to encourage their appearance. In this survey, we discuss various AI techniques that help in speeding up researches and assisting in the current COVID-19 crisis. Also, various learning techniques were emphasized. Cloud computing plays a vital role in virtualization since everyone is in isolation. We discuss various areas in which cloud computing can assist in concurring with this current pandemic. Consolidating enormous information and AI could prompt a significant achievement for the two patients and experts. In any case, even though we distinguished huge numbers of the main impetuses for the usage of AI in the clinical framework, the previously mentioned hindrances could likewise prevent it, particularly if the estimations of the partners are not regarded. Simulated intelligence and huge information must be incorporated and utilized in a moral way on the off chance that we need to create AI apparatuses that will be palatable for handling this pandemic. AI has been a powerful tool to distinguish early diseases due to coronavirus and helps in checking the state of the tainted patients. It can essentially improve treatment consistency and dynamic by creating valuable calculations. Computer-based intelligence isn’t just useful in the treatment of COVID-19 contaminated patients yet additionally for their appropriate medical checkups. It can follow the emergency of COVID-19 at various scales, for example, clinical and epidemiological applications. It is additionally useful to encourage the exploration of this infection by utilizing examining the accessible information. Simulated intelligence can help in creating appropriate treatment regimens, counteraction methodologies, and medication and immunization advancement. Be that as it may, the resulting variables of AI are viably used for describing the cerebral sickening patient and the typical patient, which were effectively grouped with 85% and 96% accuracy esteem by the planning and endorsement tests, independently. Likewise, the NLP was used to evacuate the periphery vein illness-related watchwords from clinical notes. In this way, a short time later they request the patients with periphery vein infirmity, which achieves over 91% precision. However, more future researches are to be carried out to guarantee that everybody imparts and works together in a manner that abstains from missing any basic focuses. Moral contemplations will assume a huge job by assisting us to go around potential snags in the reception of artificial intelligence instruments in helping the circumstance. Along these lines, the rest of the inquiries that despite everything destroys them, for example, the question of the sharing of duties, should be tended to. We should join a discussion among all partners worried, no matter what. In this way, it appears to be even more imperative to concentrate on quiet voices. Computer-based intelligence isn’t just useful in the treatment of COVID-19, nut additionally, for their appropriate medical check-ups. Future inquiries about this can be considered to follow the emergency of COVID-19 at various scales, for example, clinical, and epidemiological situation. It will be useful to encourage the examination of this infection by utilizing dissecting the accessible information. Likewise, in creating appropriate treatment, protective procedures, medication, and immunization advancement. More experiments and surveys are to be carried out concerning the big data gathered from each patient, utilizing this data can reduce the spread or if worth saying finding a solution to this pandemic. Further work will along these lines need to address their desires with the goal that the improvement of AI is to serve patients and not despite them. The techniques put forth advances medical data with an exactness of up to 90%.
